# Unraveling the Mechanisms of *Biebersteinia heterostemon* in Improving Hyperlipidemia: A Network Pharmacology, Molecular Docking, and In Vitro Validation in HepG2 Cells

**DOI:** 10.3390/plants14223535

**Published:** 2025-11-19

**Authors:** Xiuxiu Shen, Shengwen Chen, Mengting Zeng, Benyin Zhang

**Affiliations:** College of Eco-Environmental Engineering, Qinghai University, Xining 810016, China; ys230860020526@qhu.edu.cn (X.S.); ys230860020524@qhu.edu.cn (S.C.); ys230901j10563@qhu.edu.cn (M.Z.)

**Keywords:** *Biebersteinia heterostemon* Maxim., hyperlipidemia, network pharmacology, molecular docking, lipid metabolism

## Abstract

*Biebersteinia heterostemon* is a traditional Tibetan medicine known for its antioxidant, hypoglycemic, and anti-atherosclerotic properties. However, its therapeutic effects and mechanisms in the treatment of hyperlipidemia remain unclear. In this study, the ethyl acetate extract of *B. heterostemon* (BHEE) was first identified as the most effective lipid-lowering fraction through its inhibitory activity on pancreatic lipase and cholesterol esterase. Chemical characterization of BHEE by UHPLC-MS/MS revealed 108 compounds. Network pharmacology and molecular docking analyses were then employed to predict key active components and signaling pathways involved in BHEE’s lipid-lowering effects. A total of 50 active components and 623 targets were selected from the PubChem, SwissADME, and Swiss Target Prediction databases. These targets were intersected with 1606 hyperlipidemia-related targets from GeneCards, OMIM, and DrugBank, resulting in 144 common targets. The “drug-active component-intersecting target-pathway-HLP” and protein–protein interaction (PPI) networks suggested key active components such as 6-methoxytricin, vulgarin, flazin, ganhuangenin, and eupatorin, and core targets including TNF, IL6, AKT1, PPARG, and EGFR. GO and KEGG pathway enrichment analysis highlighted potential signaling pathways, such as AGE-RAGE, PPAR, insulin resistance, TNF, and lipid and atherosclerosis pathways. Molecular docking further predicted the strong binding affinity between key active components and core targets. At the cellular level, BHEE dose-dependently reduced lipid accumulation in FFA-induced HepG2 cells and improved oxidative stress (CAT, GSH, SOD, MDA) and inflammation (TNF-α, IL-6) markers. In conclusion, BHEE may exert its anti-hyperlipidemic effects through modulation of key targets like TNF, IL6, AKT1, PPARG, and EGFR. These findings suggest a multi-target mechanism, though further experimental validation is necessary to confirm these effects. This study provides valuable insights into the potential application of *B. heterostemon* as a natural therapeutic agent for hyperlipidemia.

## 1. Introduction

Hyperlipidemia (HLP) is a common chronic disease caused by abnormalities in lipid metabolism or transport, and is a lipid metabolic disorder [1,2]. It is typically characterized by elevated levels of total cholesterol (TC), triglycerides (TG), and low-density lipoprotein cholesterol (LDL-C), and/or reduced high-density lipoprotein cholesterol (HDL-C) levels in the serum [3]. The primary pathogenesis of HLP involves increased lipoprotein synthesis and decreased lipid consumption, resulting in abnormal elevations in lipid or lipoprotein levels in the blood [4]. Furthermore, HLP is a significant risk factor for diseases such as stroke, myocardial infarction, and atherosclerosis [5]. Currently, statins are the main drugs used to treat HLP in clinical practice, but they are often associated with adverse reactions such as liver damage and muscle pain [6,7]. Natural medicines are known for their low toxicity and diverse pharmacological activities, offering good effectiveness and safety in the prevention and treatment of diseases [8]. Research has shown that many natural substances can regulate lipid metabolism and improve hyperlipidemia. For example, hawthorn pectin can reduce the weight of hyperlipidemic mice, alleviate pathological damage to the ileum and liver, lower inflammation factors, improve antioxidant capacity, and regulate blood lipids [9]. Aqueous extract of fermented *Eucommia ulmoides* leaves has been shown to effectively lower blood lipid levels in hyperlipidemic rats by downregulating the expression of the adipogenesis gene SREBP-1c [10]. *Polygonum cuspidatum* extracts can improve blood lipid levels in hyperlipidemic rats by reducing serum TC, TG, ox-LDL, and LDL-C levels [11]. These studies suggest that plant-derived natural products have potential applications in the prevention and treatment of hyperlipidemia.

In recent years, with the rapid development of systems biology, network pharmacology and molecular docking techniques have been applied to drug development and target analysis, becoming important methods for elucidating the potential mechanisms of traditional Chinese medicine (TCM) involving multiple components, targets, and pathways [12]. These methods provide an efficient approach for pharmacological research. Network pharmacology integrates systems biology, multi-omics analysis, and computational biology approaches to predict, at the molecular level, the biological mechanisms underlying the therapeutic effects of traditional Chinese medicines on complex diseases. Through the construction of interaction networks between drug molecules and biological molecules, network pharmacology helps to discover new drug targets, understand the interaction patterns between drug molecules and targets, and predict the efficacy and toxicity of drugs [13]. Molecular docking, as a key computational tool supporting the network pharmacology framework, enables precise prediction of the binding modes and affinities between drug ligands and target proteins through computational simulations. Within the multifaceted research system of traditional medicine, this technique is indispensable for elucidating the interactions between complex herbal constituents and biomolecular targets, as well as for uncovering their underlying pharmacological effects [14]. For example, Chu et al. [15] used network pharmacology combined with molecular docking and molecular dynamics simulations to find that lentinan improves hyperlipidemia by regulating targets such as PPARs and SREBP, promoting fatty acid oxidation and inhibiting lipid synthesis. Huang et al. [16] combined network pharmacology, molecular docking, and metabolomics to discover that *Abrus mollis* Hance improves lipid metabolism disorders induced by a high-fat diet by inhibiting the PI3K-Akt signaling pathway, downregulating adipogenesis mediated by SREBP-1, and suppressing NF-κB-driven inflammation.

*Biebersteinia heterostemon* Maxim., a plant belonging to the *Biebersteinia* genus in the Biebersteiniaceae family, is mainly distributed in regions of Qinghai, Gansu, Ningxia, Sichuan, and Tibet in China, at altitudes ranging from 1000 to 3200 m. It possesses a variety of biological activities, such as analgesic, anti-inflammatory, antioxidant, hypoglycemic, and anti-atherosclerotic effects. Flavonoids, alkaloids, phenylpropanoids, terpenes, and volatile oils have been isolated and identified from *B. heterostemon* [17]. However, its lipid-lowering effects and underlying mechanisms are not yet clear. In this study, based on the evaluation of the inhibitory activities of *B. heterostemon* on pancreatic lipase and cholesterol esterase, we characterized the chemical components of the active ethyl acetate extract of *B. heterostemon* (BHEE) using UHPLC-MS/MS. Combining network pharmacology and molecular docking simulations, we aim to identify the key active ingredients and targets, as well as the mechanisms responsible for its improvement of hyperlipidemia. Additionally, the anti-hyperlipidemic effects of *B. heterostemon* will be further verified through a fatty degeneration cell model induced by free fatty acids and treated with BHEE.

## 2. Results

### 2.1. Ethyl Acetate Extract of B. heterostemon (BHEE) Exhibits the Strongest Inhibition of Pancreatic Lipase and Cholesterol Esterase

**Figure 1:**
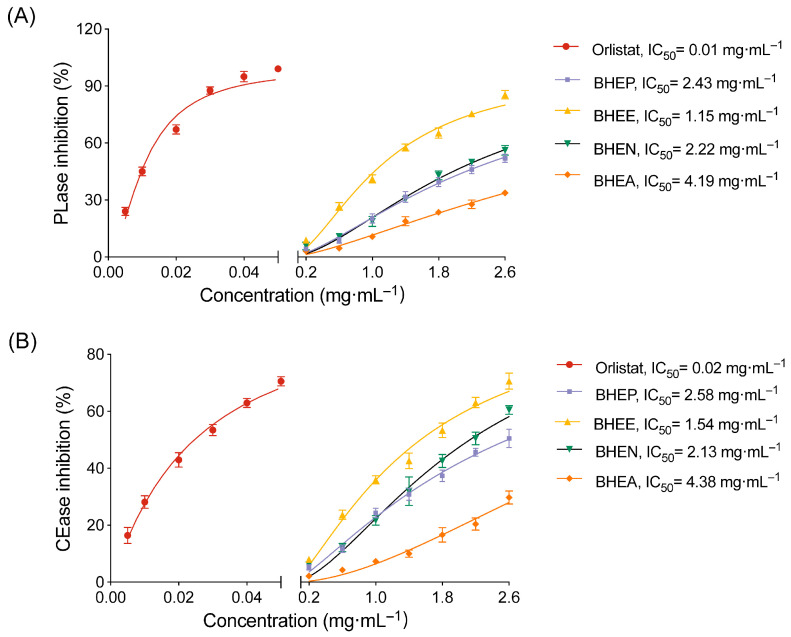
Inhibition of pancreatic lipase (PLase) and cholesterol esterase (CEase) by different extracts of *B. heterostemon*. (**A**) Inhibition of PLase activity; (**B**) Inhibition of CEase activity. BHEP, BHEE, BHEN, and BHEA represent the petroleum ether, ethyl acetate, n-butanol, and water extracts of the ethanol extract of *B. heterostemon*, respectively. Data are presented as mean ± standard deviation (n = 3).

To determine the optimal active part of *B. heterostemon* for inhibiting pancreatic lipase (PLase) and cholesterol esterase (CEase), we measured the inhibitory activities of BHEP, BHEE, BHEN and BHEA of *B. heterostemon* against these two enzymes, using orlistat as a positive control. The results indicated that all four extracts exhibited varying degrees of inhibitory effects, showing a dose-dependent response (Figure 1). Among them, the BHEE displayed the strongest inhibitory activity, with IC_50_ values of 1.15 mg·mL^−1^ for PLase and 1.54 mg·mL^−1^ for CEase. Particularly at a dose of 2.6 mg·mL^−1^, the inhibition rates for PLase and CEase reached 85.1% and 69.33%, respectively. The next strongest activities were observed for BHEN (IC_50_ = 2.22 mg·mL^−1^ and 2.13 mg·mL^−1^), BHEP (IC_50_ = 2.43 mg·mL^−1^ and 2.58 mg·mL^−1^), and BHEA (IC_50_ = 4.19 mg·mL^−1^ and 4.38 mg·mL^−1^). These findings suggest that BHEE may be the main active part responsible for its lipid-lowering effects.

Based on the in vitro enzyme inhibition results, BHEE exhibited the strongest inhibitory activity against both PLase and CEase, with the lowest IC_50_ values compared to the other three extracts. This suggests that BHEE is the key active fraction of *B. heterostemon* responsible for its lipid-lowering effects. Therefore, to further explore its pharmacological mechanisms, we have selected BHEE as the representative active extract for subsequent studies, including chemical analysis, network pharmacology, and cellular validation experiments.

### 2.2. Chemical Composition Analysis of BHEE

This study further analyzed the chemical constituents of BHEE using UHPLC-Q-Exactive-Orbitrap-MS/MS in both positive and negative ion modes (Figure S1). Through comparison with data from the TCM database and other relevant databases, a total of 108 chemical components were identified, including 44 flavonoids, 15 phenylpropanoids, 4 alkaloids, 13 terpenoids, 9 sugars and glycosides, 7 organic acids and their derivatives, 1 indole and its derivatives, 1 quinone, 6 fatty acyl compounds, and 8 other compounds (Table S1).

### 2.3. Network Pharmacology Analysis of BHEE in the Treatment of Hyperlipidemia

#### 2.3.1. Screening of Active Components of BHEE and Prediction of Potential Targets for Treating HLP

**Figure 2:**
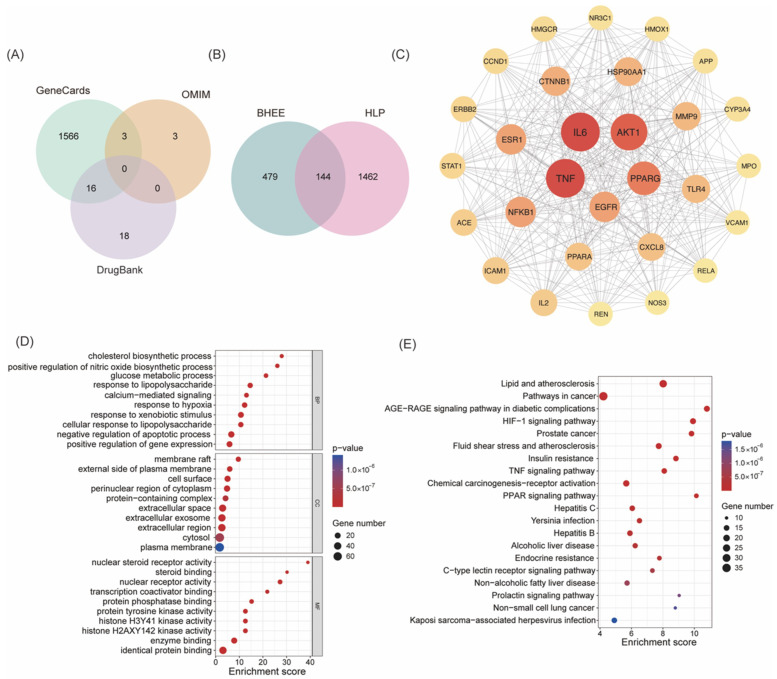
Network pharmacology analysis of BHEE in improving hyperlipidemia. (**A**) Venn diagram of hyperlipidemia targets. (**B**) Venn diagram of intersecting targets between BHEE and hyperlipidemia. (**C**) Protein–protein interaction (PPI) network of the 29 core targets filtered based on Degree > 2× median (39), Betweenness Centrality > median (44.68), and Closeness Centrality > median (0.49). (**D**) GO functional annotation of intersecting targets. (**E**) KEGG pathway enrichment analysis of intersecting targets.

Based on the principles of drug absorption, which include a gastrointestinal absorption (GI absorption) score of “high” and meeting at least two of the five drug-likeness conditions, 56 chemical components from the characterized constituents were selected (Table S2). Further target prediction excluded components without predicted targets and those with a “Probability” of 0. A total of 50 active components (Table 1) and 623 corresponding target proteins were obtained (Table S3). By searching the GeneCards, OMIM, and DrugBank databases, 1606 HLP targets were identified (Figure 2A and Table S4). The intersection of the active components’ target proteins and the HLP disease targets resulted in 144 potential targets for BHEE’s action in improving hyperlipidemia (Figure 2B and Table S5).

#### 2.3.2. Construction of Protein–Protein Interaction (PPI) Network and Screening of Key Targets

To further explore the potential mechanism of BHEE in treating HLP, a PPI network was constructed based on the 144 intersecting targets identified above (Figure S2). This PPI network contains 144 nodes and 1795 edges, with an average node degree of 24.9, indicating that there are extensive interactions among the targets and a high level of network connectivity. The PPI results were imported into Cytoscape v3.9.1 software for visualization, generating a network diagram. To further identify the key active components, topological analysis of the network was performed using the CytoNCA plugin (Table S6). Based on Degree Centrality, Betweenness Centrality, and Closeness Centrality, 29 core targets were selected (Figure 2C and Table 2). These 29 core targets were ranked by their Degree values from high to low, with the top 10 key targets being: Tumor necrosis factor (TNF), Interleukin-6 (IL-6), Serine/threonine-protein kinase B1 (AKT1), Peroxisome proliferator-activated receptor γ (PPARG), Epidermal growth factor receptor (EGFR), Nuclear factor of kappa light polypeptide gene enhancer in B-cells 1 (NF-κB1), Estrogen receptor 1 (ESR1), Catenin (Cadherin-Associated Protein), beta 1 (CTNNB1), Heat shock protein HSP 90-alpha (HSP90AA1), and Recombinant Matrix Metalloproteinase 9 (MMP9). These targets play crucial roles in various biological processes, including inflammatory response, lipid metabolism, insulin signaling pathways, and cell proliferation regulation. This suggests that BHEE may exert its anti-HLP effects by synergistically regulating these pathways through multiple targets.

#### 2.3.3. GO Functional and KEGG Metabolic Pathway Enrichment Analysis

In this study, GO functional enrichment analysis and KEGG pathway enrichment analysis were further performed on the 144 intersecting targets of active components and diseases. The results revealed that 709 significant enriched terms were obtained from the GO functional enrichment (Table S7), covering three categories: Biological Process (BP) with 481 terms, Cellular Component (CC) with 64 terms, and Molecular Function (MF) with 164 terms. To visually display the results, the top 10 significant terms in each category were processed using the online platform Bioinformatics (Figure 2D). The analysis showed that the potential effects of BHEE may primarily occur in cellular structural regions such as membrane rafts, the external side of the plasma membrane, and the cell surface. At the biological process level, the terms were significantly enriched in key physiological and pathological processes, including cholesterol biosynthesis, positive regulation of nitric oxide (NO) biosynthesis, glucose metabolic processes, and responses to lipopolysaccharide (LPS). In terms of molecular function, the main functions involved steroid binding, nuclear steroid receptor activity, and transcription coactivator binding.

The KEGG pathway enrichment analysis revealed that the 144 intersecting targets were enriched in 156 signaling pathways (Table S8). The top 10 pathways with the highest enrichment significance were selected for visualization (Figure 2E). The results indicate that the anti-HLP effects of BHEE are closely related to multiple the key signaling pathways, primarily including the AGE-RAGE signaling pathway, PPAR signaling pathway, HIF-1 signaling pathway, insulin resistance pathway, TNF signaling pathway, and lipid metabolism and atherosclerosis-related pathways. These pathways play crucial roles in inflammation, lipid metabolism disorders, oxidative stress, and insulin sensitivity regulation, further supporting the potential mechanism by which BHEE synergistically intervenes in the pathological progression of HLP through multiple pathways.

#### 2.3.4. Construction of the “Drug-Effective Component-Intersecting Target-Pathway” Network

To systematically explore the potential mechanism of BHEE in treating HLP, a “Drug-Effective Component-Intersecting Target-Pathway” network was constructed using Cytoscape v3.9.1 software to reveal its key active components and the interactions between these components and their targets. The network analysis revealed that the network contains 216 nodes and 1314 edges, demonstrating a dense structure. This suggests that BHEE may exert its anti-hyperlipidemic effects through a multi-component and multi-target synergistic mechanism. However, the precise interactions among these components require further investigation.

**Figure 3:**
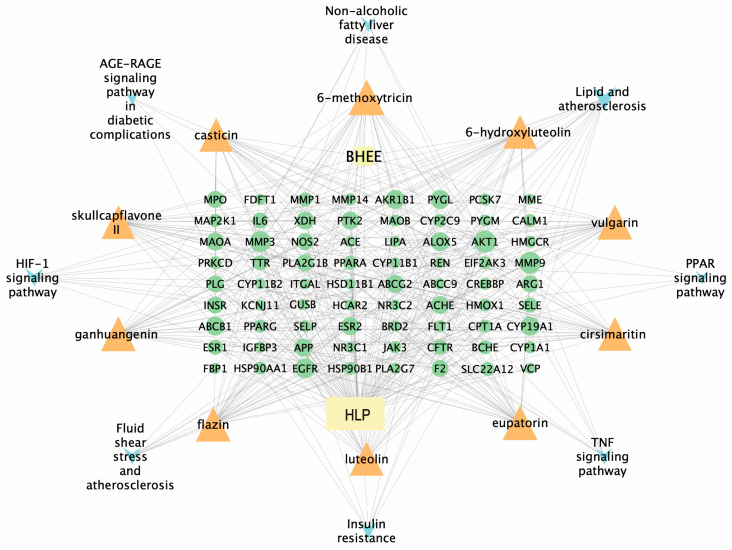
“Drug-Key Active Components-Intersecting Target-Pathway” Network. Rectangle nodes represent the BHEE or hyperlipidemia, triangle nodes represent key active components, circular nodes represent intersecting targets corresponding to key active ingredients, and “V” nodes represent related pathways.

To further identify the key active components, a topological analysis of the network was conducted using the CytoNCA plugin in Cytoscape v3.9.1 software, calculating the Degree Centrality of each node (Table S9). Based on the ranking of Degree Centrality, the top 10 potential key active components were selected (Table 3). These included eight flavonoid components: 6-methoxytricin, ganhuangenin, eupatorin, luteolin, casticin, cirsimaritin, skullcapflavone II, and 6-hydroxyluteolin; one terpenoid component, vulgarin; and one alkaloid component, flazin. The corresponding targets and related pathways were visualized (Figure 3). These results suggest that flavonoid components dominate the network in BHEE’s treatment of hyperlipidemia (HLP), exhibiting high topological significance. This indicates that flavonoids may play a central role as the core active substances responsible for BHEE’s anti-HLP effects, providing an important reference for future research on its pharmacodynamic material basis.

### 2.4. Molecular Docking of Potential Key Components and Targets

**Figure 4:**
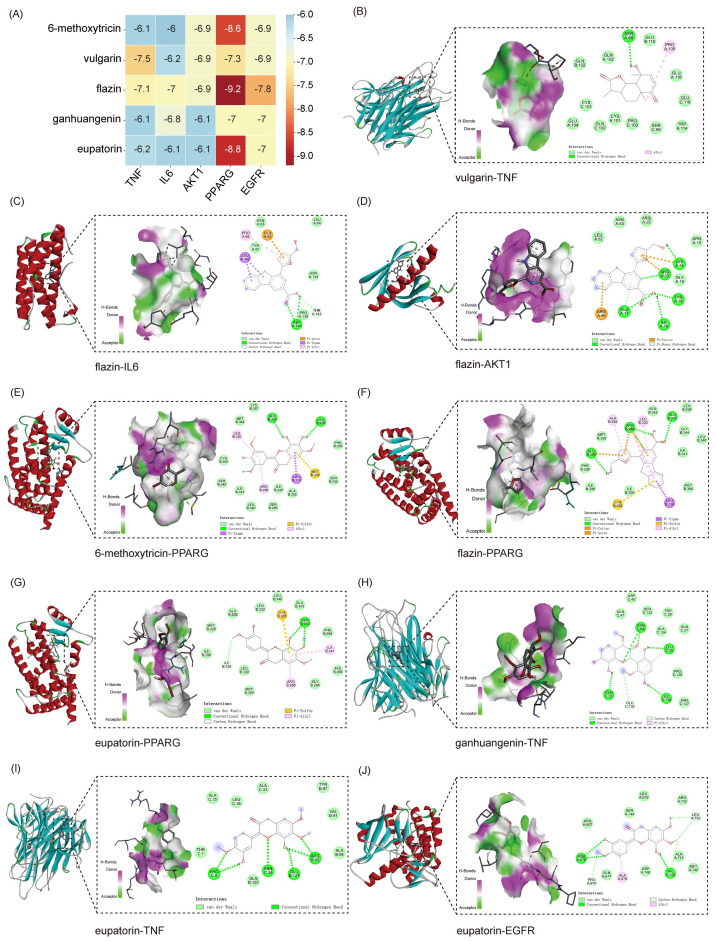
Molecular docking analysis of key active components and core targets of BHEE in improving hyperlipidemia. (**A**) Heatmap of molecular binding energies. (**B**) Docking of vulgarin with TNF. (**C**) Docking of flazin with IL6. (**D**) Docking of flazin with AKT1. (**E**) Docking of 6-methoxytricin with PPARG. (**F**) Docking of flazin with PPARG. (**G**) Docking of eupatorin with PPARG. (**H**) Docking of ganhuangenin with TNF. (**I**) Docking of eupatorin with TNF. (**J**) Docking of eupatorin with EGFR.

To further validate the interaction potential between the active components of BHEE and key targets, molecular docking simulations were conducted to explore their binding modes and affinities. Degree Centrality is a core metric for assessing the importance of network nodes, such as drugs, components, and targets [18]. Components with high Degree Centrality are likely to be core ingredients responsible for the therapeutic effects of the drug in treating diseases. The top five core targets identified from the PPI network—TNF, IL6, AKT1, PPARG, and EGFR—were selected for docking with the top five potential key active components identified in the “Drug-Effective Component-Intersecting Target-Pathway” network: 6-methoxytricin, vulgarin, flazin, ganhuangenin, and eupatorine (Figure 4 and Figure S3).

The binding energy obtained from molecular docking serves as a key indicator for evaluating the strength of interactions between a ligand and its protein receptor, and it is typically expressed as a negative value [19]. When the binding free energy between a ligand and a receptor protein is lower than −5.0 kcal·mol^−1^, it indicates stable binding, and the more negative the value, the stronger the binding affinity [19,20]. The docking results revealed that all five active components exhibited binding free energies less than −5.0 kcal·mol^−1^ with the five core targets, indicating a strong potential for interaction between BHEE’s potential key components and HLP-related targets. Further analysis of the key binding conformations showed that vulgarin forms four hydrogen bonds with the TNF protein at specific residues, resulting in a binding energy of −7.5 kcal/mol, which indicates a strong interaction. Flazin interacts with AKT1, forming five hydrogen bonds at specific residues, with a binding energy of −6.9 kcal/mol. It also binds with PPARG, forming four hydrogen bonds, with a much higher binding affinity of −9.2 kcal/mol. Ganhuangenin forms six hydrogen bonds with TNF at various residues, with a binding energy of −6.1 kcal/mol. Eupatorin also interacts with TNF, forming six hydrogen bonds, and binds with EGFR, resulting in binding energies of −6.2 kcal/mol and −7.0 kcal/mol, respectively.

These findings show that multiple active components form stable interactions with key targets, and the generally low binding energies suggest that they have good spatial matching and strong binding abilities with the target proteins. The molecular docking results further support the hypothesis that the potential key active components in BHEE can effectively bind to core HLP-related target proteins, providing a solid theoretical foundation for subsequent pharmacological studies and mechanism validation. This supports the view that BHEE may exerts its effects in improving hyperlipidemia through a multi-component, multi-target synergistic approach.

### 2.5. Effect of Different Concentrations of BHEE on HepG2 Cell Metabolic Activity

**Figure 5:**
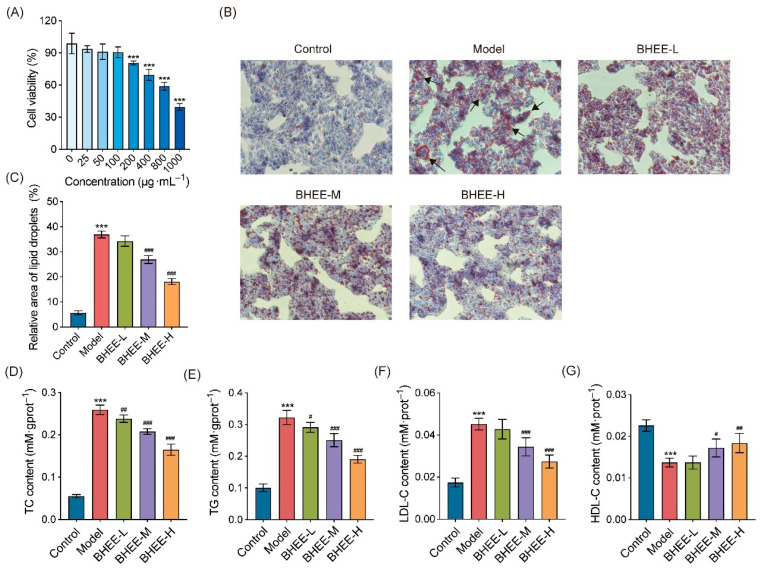
Effect of BHEE on HepG2 Cell Viability and Lipid Levels in FFA-Induced HepG2 Cells. (**A**) HepG2 cell viability after treatment with different concentrations of BHEE (0, 25, 50, 100, 200, 400, 800, 1000 μg·mL^−1^). (**B**) Representative images of Oil Red O staining (100× magnification). The black arrow refers to the accumulated lipid droplets. (**C**) Relative lipid droplet area. Levels of (**D**) TC, (**E**) TG, (**F**) LDL-C, and (**G**) HDL-C in HepG2 cells. Control: Normal control; Model: HepG2 cells treated with FFA for 24 h; BHEE-L: HepG2 cells treated with FFA and 25 μg·mL^−1^ BHEE for 24 h; BHEE-M: HepG2 cells treated with FFA and 50 μg·mL^−1^ BHEE for 24 h; BHEE-H: HepG2 cells treated with FFA and 100 μg·mL^−1^ BHEE for 24 h. Data are presented as mean ± standard deviation (n = 3). *** *p* < 0.001 vs. Control; ^#^ *p* < 0.05, ^##^ *p* < 0.01, ^###^ *p* < 0.001 vs. Model.

This study further explored the regulatory effect of BHEE on lipid accumulation at the cellular level. First, the CCK-8 assay was used to evaluate the effects of different concentrations of BHEE (0, 25, 50, 100, 200, 400, 800, 1000 μg·mL^−1^) on the metabolic activity of HepG2 cells (Figure 5A). When BHEE concentrations were 25, 50, and 100 μg·mL^−1^, there was no significant difference in HepG2 cell metabolic activity compared to the control group (*p* > 0.05), indicating that BHEE had no noticeable inhibitory effect on cell growth within this concentration range. However, as the concentration of BHEE increased, cell metabolic activity decreased, suggests that high concentrations of BHEE have a certain effect on the viability of HepG2 cells. Therefore, concentrations of 25, 50, and 100 μg·mL^−1^ were selected for subsequent experiments, and were defined as the low-dose group (BHEE-L), medium-dose group (BHEE-M), and high-dose group (BHEE-H).

### 2.6. Effect of BHEE on Lipid Content in Free Fatty Acid (FFA)-Induced HepG2 Cells

This study used an FFA-induced HepG2 cell model to induce lipid deposition, and Oil Red O staining was employed to observe lipid accumulation (Figure 5B,C). Compared to the control group, the model group exhibited a significant accumulation of red lipid droplets within the cells, indicating a marked increase in lipid deposition, thus confirming the successful establishment of the lipid deposition cell model. After treatment with different concentrations of BHEE, lipid droplet accumulation was notably reduced, with both the number and area of lipid droplets decreasing in a dose-dependent manner. Additionally, the volume of lipid droplets became smaller. Notably, in the high-dose BHEE group (BHEE-H), the area of lipid droplets was reduced by 50.97%, demonstrating a significant lipid clearance effect. These results suggest that BHEE can effectively alleviate FFA-induced lipid accumulation in HepG2 cells in a dose-dependent manner. Further analysis revealed the effect of BHEE on the levels of TC, TG, LDL-C, and HDL-C in FFA-induced HepG2 cells (Figure 5D–G). The results showed that compared to the normal control group, FFA treatment significantly increased the levels of TC, TG, and LDL-C while decreasing HDL-C levels. However, BHEE treatment significantly reversed this abnormal metabolic state. In the BHEE-L group (25 μg·mL^−1^), TC and TG levels decreased by 8.20% and 9.79%, respectively; in the BHEE-M group (50 μg·mL^−1^), TC, TG, and LDL-C levels decreased by 19.85%, 22.24%, and 23.96%, respectively, while HDL-C levels increased by 25.55%; in the BHEE-H group (100 μg·mL^−1^), TC, TG, and LDL-C levels further decreased by 36.40%, 40.94%, and 39.56%, respectively, and HDL-C levels increased by 34.31%. These improvements in lipid profile markers exhibited a dose-dependent trend, further confirming the positive role of BHEE in regulating lipid metabolism disorders in cells.

### 2.7. Effect of BHEE on Antioxidant Capacity in FFA-Induced HepG2 Cells

**Figure 6:**
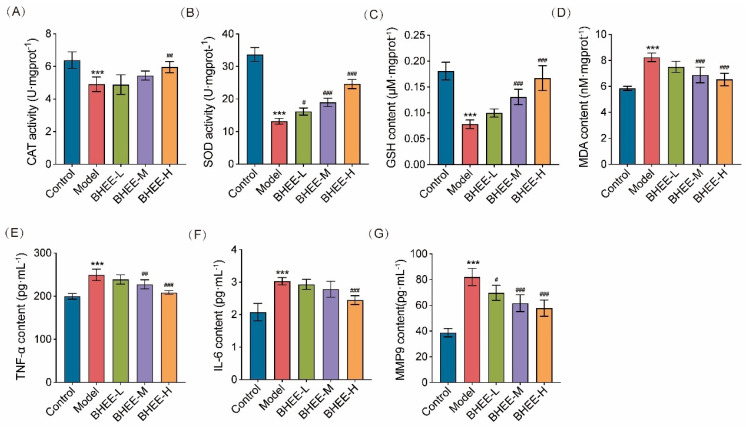
Effect of BHEE on Oxidative Stress and Inflammation in FFA-Induced HepG2 Cells. Levels of (**A**) CAT, (**B**) SOD, (**C**) GSH, (**D**) MDA, (**E**) TNF-α, (**F**) IL-6, and (**G**) MMP9 in HepG2 cells. Control: Normal control; Model: HepG2 cells treated with FFA for 24 h; BHEE-L: HepG2 cells treated with FFA and 25 μg·mL^−1^ BHEE for 24 h; BHEE-M: HepG2 cells treated with FFA and 50 μg·mL^−1^ BHEE for 24 h; BHEE-H: HepG2 cells treated with FFA and 100 μg·mL^−1^ BHEE for 24 h. Data are presented as mean ± standard deviation (n = 3). *** *p* < 0.001 vs. Control; ^#^ *p* < 0.05, ^##^ *p* < 0.01, ^###^ *p* < 0.001 vs. Model.

To investigate whether BHEE plays a role in maintaining redox homeostasis in FFA-induced HepG2 cells, we measured the effects of BHEE on intracellular CAT and SOD activity, GSH levels, and MDA content (Figure 6A–D). The results showed that compared to the normal control group, the model group had significantly reduced GSH levels and CAT and SOD enzyme activities (*p* < 0.001), while MDA levels were significantly elevated (*p* < 0.001), indicating a marked oxidative stress response induced by FFA. After treatment with BHEE, all of these indicators were significantly improved in a dose-dependent manner. Notably, in the high-dose group (BHEE-H, 100 μg·mL^−1^), the antioxidant capacity of HepG2 cells was significantly enhanced, with GSH levels, CAT activity, and SOD activity increasing by 113.26%, 21.76%, and 86.66%, respectively, while MDA levels decreased by 20.62%. These results indicate that BHEE can effectively enhance the levels of endogenous antioxidants and key antioxidant enzyme activities in cells, inhibiting lipid peroxidation and reducing oxidative damage caused by FFA.

### 2.8. Effect of BHEE on Inflammation in FFA-Induced HepG2 Cells

Network pharmacology analysis suggested that inflammation might play a crucial role in the improvement of HLP by BHEE. Therefore, we further measured the secretion levels of inflammation-related factors TNF-α, IL-6, and matrix metalloproteinase MMP9 to evaluate the anti-inflammatory effects of BHEE on FFA-induced inflammation. The results (Figure 6E–G) showed that, compared to the normal control group, the model group exhibited significantly elevated levels of TNF-α, IL-6, and MMP9 (*p* < 0.001), indicating a chronic inflammatory state induced by lipid deposition. After treatment with different concentrations of BHEE, the secretion levels of these inflammatory factors decreased in a dose-dependent manner. Specifically, the BHEE-L group (25 μg·mL^−1^) reduced MMP9 levels by 15.02%; the BHEE-M group (50 μg·mL^−1^) decreased TNF-α and MMP9 levels by 8.80% and 24.82%, respectively; and the BHEE-H group (100 μg·mL^−1^) significantly suppressed the release of multiple inflammatory factors, with TNF-α, IL-6, and MMP9 levels decreasing by 16.40%, 19.10%, and 29.55%, respectively. These results suggest that BHEE effectively inhibits the secretion of inflammatory factors induced by FFA, alleviates the inflammatory response in cells, and that its anti-inflammatory effects increase with concentration. This indicates that BHEE may alleviate chronic low-grade inflammation associated with a high-fat environment through the regulation of inflammatory pathways, providing protection against hyperlipidemia.

## 3. Discussion

Hyperlipidemia is a common chronic condition characterized by disturbances in lipid metabolism and has become a major risk factor for human health. Elevated blood lipid levels in patients with hyperlipidemia trigger chronic inflammation, which in turn enhances oxidative stress and aggravates lipid peroxidation [21,22]. These processes significantly increase the risk of developing metabolic diseases such as cardiovascular disease, hypertension, fatty liver, and diabetes [23,24,25]. Thus, effective prevention and treatment of hyperlipidemia are of great importance for improving public health. In this study, by integrating network pharmacology, molecular docking, and cellular experiments, we systematically investigated the potential bioactive components, targets, and signaling pathways of BHEE in alleviating hyperlipidemia and provided preliminary experimental validation of the predicted mechanisms.

PLase and CEase are key regulators of lipid digestion and absorption, and their inhibition can effectively reduce dietary fat uptake, thereby improving hyperlipidemia [26]. Our findings first demonstrated that BHEE exerted the most significant inhibitory effects on both enzymes compared to other extracts such as BHEP, BHEN, and BHEA, suggesting its potential to modulate lipid metabolism. However, the precise inhibition mechanism—whether competitive, non-competitive, or mixed—was not investigated. Future enzyme kinetics experiments will be performed to clarify the mode of inhibition and to integrate these findings with molecular docking analyses for a more complete understanding of BHEE’s enzyme interaction mechanism. Nevertheless, this result highlights the extract with the greatest potential, offering valuable insights for further research into its anti-hyperlipidemic potential. In addition, Figure 1 demonstrates that the inhibitory activity of the BHEE extracts on pancreatic lipase and cholesterol esterase is lower than that of the positive control, Orlistat. Orlistat, a potent synthetic inhibitor, irreversibly binds to pancreatic lipase and is highly effective in reducing the digestion and absorption of triglycerides [27]. In comparison, BHEE contains a variety of bioactive compounds, which, although effective, may not exhibit the same level of potency as Orlistat in terms of enzyme inhibition. Natural extracts, such as BHEE, typically exert their effects through a more complex, multi-target approach, often promoting gradual metabolic improvements rather than immediate, high-potency inhibition. Thus, while the extracts show lower potency than Orlistat in isolated enzyme assays, their potential benefits may lie in their ability to modulate broader metabolic pathways and support long-term lipid balance.

While our enzyme inhibition assays indicated relatively high IC_50_ values for PLase and CEase, the effective concentrations required to observe lipid-lowering effects in HepG2 cells were significantly lower. This discrepancy may be explained by several factors, including the bioavailability of BHEE and its intracellular distribution, which could allow for more efficient targeting of the enzymes at the cellular level. Additionally, synergistic interactions among the bioactive components of BHEE may enhance its overall effectiveness at lower concentrations. These considerations highlight the importance of further studies to assess the pharmacokinetics and bioavailability of BHEE, as well as the potential synergistic effects of its compounds in cellular models.

UHPLC-MS/MS analysis identified 108 compounds in BHEE. By integrating data from SwissADME, TCMSP, and Lipinski’s rule of five, 50 candidate active components were screened, indicating that BHEE likely acts through multiple components. The constructed “drug–active component–target-pathway” network highlighted several compounds as potential core bioactive molecules, including 6-methoxytricin, vulgarin, flazin, ganhuangenin, and eupatorin. Previous studies have confirmed their biological activities in regulating metabolism. For example, flazin reduces lipid accumulation in HK-2 cells by inhibiting adipogenesis, promoting lipolysis, and lowering TG levels, while also protecting against oxidative stress by downregulating ROS and mitochondrial apoptotic signaling [28,29]. Ganhuangenin, a natural antioxidant, inhibits the formation of phosphatidylcholine hydroperoxide and oxidized LDL, displaying strong anti-lipid peroxidation and tissue-protective effects [30,31]. Eupatorin shows potent anti-inflammatory and antioxidant activities by suppressing the release of NO, TNF-α, and IL-6 in LPS-stimulated macrophages [32]. These reports suggest that the therapeutic effects of BHEE against hyperlipidemia may derive from its multi-component regulation of lipid metabolism, oxidative stress and inflammatory pathways. The compounds identified as potential key active components, such as 6-methoxytricin and vulgarin, require further experimental validation. These potential bioactive molecules need to be isolated and purified in subsequent studies to evaluate their individual therapeutic potential and determine whether they act synergistically or independently in the observed effects.

Hyperlipidemia is closely associated with inflammation and oxidative stress. Elevated pro-inflammatory cytokines such as TNF-α and IL-6 have been linked to lipid metabolic disorders, while oxidative stress biomarkers including GSH, CAT, SOD, and MDA are widely recognized indicators of redox homeostasis [11,33]. Additionally, MMP9 is considered a downstream mediator of inflammatory and matrix remodeling processes, which are functionally linked to lipid metabolism and hepatic injury in hyperlipidemia [34]. In our FFA-induced HepG2 model, BHEE treatment restored antioxidant enzyme activity, increased GSH, reduced MDA, and suppressed pro-inflammatory mediators TNF-α, IL-6, and MMP9 in a dose-dependent manner. Similarly, berberine is a naturally occurring isoquinoline alkaloid found in *Coptis chinensis* and several other herbal extracts, and it has been shown to effectively improve hyperlipidemia [35]. Studies have demonstrated that berberine enhances lipid metabolism, lowers LDL-C, TG, and TC levels, improves insulin resistance and oxidative stress, and exerts anti-inflammatory effects through the inhibition of TNF-α and IL-6 secretion, as well as modulation of the PPARγ signaling pathway [36]. These findings indicate that BHEE not only regulates lipid metabolism but also alleviates oxidative injury and chronic inflammation caused by lipid overload, providing experimental confirmation of the network pharmacology predictions. In addition, although the FFA-induced HepG2 steatosis model is a widely accepted and reproducible system for studying lipid metabolism, oxidative stress, and inflammatory responses in hepatocytes, it primarily reflects hepatic dysfunction and cannot fully represent the systemic metabolic alterations associated with hyperlipidemia. Nevertheless, this model has been extensively used in related research—for example, Zeng et al. [37] and Chu et al. [15] both employed FFA- or oleic acid-induced HepG2 models to investigate the lipid-lowering effects of natural compounds on hyperlipidemia. Therefore, the current findings should be considered preliminary, and further in vivo studies are warranted to confirm the comprehensive metabolic effects of BHEE on multiple organs involved in lipid regulation.

PPI network analysis identified TNF, IL6, AKT1, PPARG, EGFR, and MMP9 as key targets in the anti-hyperlipidemic effects of BHEE. TNF and IL-6 are central inflammatory cytokines implicated in lipid metabolism disorders and insulin resistance [38,39]. AKT1, a serine/threonine kinase downstream of PI3K, plays a crucial role in glucose and lipid homeostasis by phosphorylating metabolic regulators [40]. PPARG, a nuclear receptor, regulates adipogenesis, insulin sensitivity, and lipid clearance by modulating LDL receptor expression and activity [41]. MMP9, once mainly studied in vascular remodeling and atherosclerosis, is increasingly recognized for its role in cholesterol metabolism and lipid homeostasis [42]. Molecular docking confirmed that the potential key components of BHEE exhibited stable binding with these targets, reflecting strong molecular affinity and supporting their involvement as critical nodes in BHEE’s pharmacological activity. Cell-based experiments further validated these findings, demonstrating that BHEE significantly reduced TNF-α, IL-6, and MMP9 secretion in FFA-stimulated HepG2 cells. Other key targets, including AKT1, PPARG, and EGFR, were identified through network pharmacology and molecular docking analyses but were not directly validated in this study. These targets will be the focus of future in vitro and in vivo investigations. It is important to note that the mechanistic insights derived from network pharmacology and molecular docking are predictive in nature and require experimental validation. While we have proposed potential targets, such as TNF, AKT1, and PPARG, based on these in silico approaches, further experimental studies (e.g., Western blotting, qPCR) will be necessary to confirm the modulation of these targets by BHEE in the cell model. This validation will be a key focus of our subsequent research.

KEGG enrichment analysis revealed that BHEE’s putative targets were significantly associated with signaling pathways central to lipid metabolism and inflammation, including AGE-RAGE, PPAR, HIF-1, insulin resistance, TNF, and lipid/atherosclerosis pathways. Overactivation of the AGE-RAGE pathway promotes NF-κB activation, leading to pro-inflammatory cytokine release and exacerbation of oxidative stress, thereby accelerating hyperlipidemia progression [43,44]. The PPAR signaling pathway is a master regulator of cholesterol synthesis, transport, and catabolism, playing a vital role in lipid homeostasis [45]. Insulin resistance, a hallmark of metabolic syndrome, is tightly linked to dyslipidemia and hepatic fat accumulation [46]. HIF-1 signaling, particularly under hypoxic conditions, promotes lipid uptake while inhibiting fatty acid oxidation, leading to intracellular lipid deposition [47,48]. Taken together, these findings indicate that BHEE may exerts its protective effects against hyperlipidemia through multi-target, multi-pathway regulation of lipid metabolism, oxidative stress, and inflammation.

Based on the above analyses, the active components of BHEE may exert their lipid-lowering and anti-oxidative effects by targeting key molecules such as TNF, IL-6, AKT1, PPARG, and EGFR. On one hand, they inhibit the release of pro-inflammatory cytokines like TNF-α and IL-6, blocking their role in promoting adipocyte dysfunction. On the other hand, by modulating PPARG and AKT1, BHEE restores normal insulin signaling and lipid metabolism, promoting LDL clearance and inhibiting hepatic lipogenesis. Additionally, BHEE compounds enhance antioxidant defenses and suppress ROS production, reducing oxidative stress and lipid peroxidation. Ultimately, the regulation of these key targets leads to the inhibition of lipogenesis, promotion of lipid catabolism, and alleviation of inflammation and oxidative stress, thereby improving hyperlipidemia. Although the in vitro and in silico results provide promising evidence for the lipid-lowering effects of BHEE, there are several clinical limitations to be considered. The findings from cell-based models and computational predictions may not fully reflect the complexities of human physiology. Additionally, the absence of in vivo validation and clinical studies means that the therapeutic efficacy and safety of BHEE in humans remain to be confirmed. Further clinical trials are necessary to validate the pharmacological effects and therapeutic potential of BHEE in real-world settings.

## 4. Materials and Methods

### 4.1. Plant Materials

The aerial parts of *B. heterostemon* were collected in July 2024 from Guinan County, Qinghai Province, China. The plant was identified by Prof. Benyin Zhang as *Biebersteinia heterostemon* Maxim., and the specimen (XDN-2024-0628.1) is stored at the Natural Product Laboratory of the College of Ecological Environment and Engineering, Qinghai University.

### 4.2. Sample Extraction

After air-drying, the aerial parts of *B. heterostemon* were powdered and extracted three times with 95% ethanol (solvent to material ratio of 1:15) for 8 h each time. The extract was filtered and concentrated under reduced pressure at 50 °C, yielding a crude ethanol extract weighing 4077.54 g. The extract was then suspended in water and sequentially extracted with petroleum ether, ethyl acetate, and n-butanol (1:1, organic solvent: water) to obtain the respective fractions: petroleum ether (BHEP, 231.20 g), ethyl acetate (BHEE, 243.39 g), n-butanol (BHEN, 620.35 g), and water extract (BHEA, 798.39 g). The extracts were freeze-dried and stored for further use.

### 4.3. Pancreatic Lipase and Cholesterol Esterase Inhibition Activity

The inhibitory activities of different extracts from *B. heterostemon* against pancreatic lipase (PLase, Macklin Reagent Co., Ltd., Shanghai, China, Cat. No. 9001-62-1) and cholesterol esterase (CEase, Shanghai Yuanye Biotechnology Co., Ltd., Shanghai, China, Cat. No. 9026-00-0) were assessed to identify the active lipid-lowering fractions. PLase inhibition activity was carried out according to Jiang’s method [49] with slight modifications. Lauric acid 4-nitrophenyl ester (Aladdin Reagent Co., Ltd., Shanghai, China, Cat. No. 1956-11-2) was dissolved in 5 mM sodium acetate buffer (containing 1% Triton X-100) to a final concentration of 0.8 mg·mL^−1^. Various concentrations of samples (0.2, 0.6, 1.0, 1.4, 1.8, 2.2 and 2.6 mg·mL^−1^) and pig PLase solution (2 mg·mL^−1^) were prepared. In a 96-well plate, 60 μL of the sample solution, 40 μL of lauric acid 4-nitrophenyl ester solution, and 40 μL of phosphate-buffered saline (PBS) were added. After a 10 min pre-incubation at 37 °C, 50 μL of PLase solution was added, and the reaction proceeded at 37 °C for 30 min. Absorbance at 405 nm was measured using a microplate reader (Thermo Fisher Scientific, Waltham, MA, USA). Orlistat (Macklin Reagent Co., Ltd., Shanghai, China, Cat. No. 96829-58-2) was used as a positive control. The PLase inhibition rate was calculated as follows, PLase Inhibition Rate (%) = [1 − (C − D)/(A − B)] × 100%, where A is the absorbance of the blank group (with enzyme, without sample), B is the absorbance of the control group (without enzyme, without sample), C is the experimental group absorbance (with enzyme, with sample), and D is the experimental control group absorbance (without enzyme, with sample).

CEase inhibition activity was carried out according to Zhao’s method [50] with slight modifications. p-Nitrophenyl butyrate (p-NPB, Shanghai Yuanye Biotechnology Co., Ltd., China, Cat. No. 2635-84-9) was dissolved in acetonitrile (10 mM), and CEase was dissolved in 0.1 M phosphate buffer (containing 5.16 mM sodium taurocholate and 0.1 M sodium chloride, pH 7.0) to a concentration of 20 μg·mL^−1^. 20 μL of p-NPB and 50 μL of various concentrations of BHEE samples (0.2, 0.6, 1.0, 1.4, 1.8, 2.2, 2.6 mg·mL^−1^) were mixed and incubated at 37 °C for 10 min. Then, 30 μL of CEase solution was added, and the reaction continued for 20 min. Absorbance at 405 nm was measured with a microplate reader. Orlistat was used as a positive control. The CEase inhibition rate was calculated as follows, CEase Inhibition Rate (%) = [1 − (C − D)/(A − B)] × 100%, where A is the absorbance of the blank group, B is the absorbance of the control group, C is the experimental group absorbance, and D is the experimental control group absorbance.

### 4.4. UHPLC-MS/MS Analysis of BHEE Chemical Components

A 100 mg sample of freeze-dried BHEE was dissolved in 1 mL methanol-water solution. The mixture was vortexed for 1 min, centrifuged, and diluted 20 times with methanol-water solution. 200 μL of the supernatant was analyzed. The chemical components of BHEE were analyzed using an ACQUITY UPLC I-Class HF system (Waters Corporation, Milford, MA, USA) coupled with a Q-Exactive Orbitrap high-resolution mass spectrometer (Thermo Fisher Scientific, Waltham, MA, USA).

Chromatographic conditions: The ACQUITY UPLC HSS T3 column (100 mm × 2.1 mm, 1.8 μm, Waters Corporation, Milford, MA, USA) was used for separation at a column temperature of 45 °C. The mobile phase consisted of 0.1% formic acid in water (A) and acetonitrile (B), with a gradient elution: 0–2 min, 5% B; 2–14 min, 5% to 100% B; 14–15 min, 100% B; 15–15.1 min, 100% to 5% B; 15.1–16 min, 5% B. The flow rate was 0.35 mL/min, with an injection volume of 5 μL.

Mass spectrometry conditions: The Q-Exactive Orbitrap mass spectrometer with an electrospray ionization source was used in both positive and negative ion modes. Ion source parameters: positive mode 3800 V, negative mode 3000 V. The full MS resolution was 70,000, and dd-MS2 resolution was 17,500, with a scanning range of *m*/*z* 100–1200. The auxiliary gas flow rate was 8 Arb, capillary temperature was 320 °C, and drying gas temperature was 350 °C. Data were collected in DDA mode.

The data were processed using Progenesis QI v3.0 software (Nonlinear Dynamics, Newcastle, UK) for baseline filtering, peak identification, integration, retention time calibration, peak alignment, and normalization. Compound identification was based on comparison with standards, accurate molecular weight, secondary fragmentation, and isotope distribution using the TCM database (TCMID, https://www.bidd.group/TCMID/, available at 15 March 2025).

### 4.5. Network Pharmacology Analysis

#### 4.5.1. Active Component Screening and Target Identification

The canonical SMILES of BHEE chemical components were obtained from PubChem (https://pubchem.ncbi.nlm.nih.gov/, available at 20 March 2025). These were uploaded to the SwissADME (http://www.swissadme.ch/, available at 25 March 2025) for drug-likeness prediction, and active components were selected based on criteria such as gastrointestinal absorption (GI absorption) and Lipinski’s Rule of Five. Active components were further mapped to potential targets using the SwissTargetPrediction database (http://swisstargetprediction.ch/, available at 27 March 2025).

#### 4.5.2. Hyperlipidemia Target Retrieval

Using GeneCards (https://www.genecards.org/, version 5.24 available at 2 April 2025), OMIM (https://www.omim.org/, available at 2 April 2025), and DrugBank (https://go.drugbank.com/, version 5.1.13, available at 2 April 2025), hyperlipidemia targets were obtained and consolidated. The disease targets were converted into gene names using UniProt (https://www.uniprot.org/, release 2025_01, available at 2 April 2025). A Venn diagram was generated using the online platform Bioinformatics (http://www.bioinformatics.com.cn/, available at 2 April 2025) to find the intersection of BHEE active components and hyperlipidemia targets.

#### 4.5.3. PPI Network Construction and Core Target Screening

The intersection targets were uploaded to STRING (https://cn.string-db.org/, version 12.0, available at 4 April 2025) for PPI network construction. The results were visualized in Cytoscape v3.9.1. The selection of core targets in the PPI network was based on established network pharmacology methods, where nodes with higher degree, betweenness centrality, and closeness centrality were considered most likely to be functionally important. These thresholds (Degree > 2× median, Betweenness Centrality > median, Closeness Centrality > median) were chosen to prioritize targets with the highest connectivity and centrality within the network, based on similar approaches in the literature [51].

#### 4.5.4. GO Functional and KEGG Pathway Enrichment Analysis

The intersection targets were imported into the DAVID database (https://davidbioinformatics.nih.gov/, available at 5 April 2025) for Gene Ontology (GO) functional annotation and KEGG pathway enrichment analysis. The top 10 enriched terms for Biological Process, Molecular Function, Cellular Component, and KEGG pathways were visualized using the online platform Bioinformatics (http://www.bioinformatics.com.cn/, available at 5 April 2025).

#### 4.5.5. Construction of “Drug-Active Component-Target-Pathway” Network

The drug-active component-target-pathway network was constructed using Cytoscape v3.9.1, incorporating active components, intersection targets, and the top 20 enriched pathways. The CytoNCA plugin was used for topological analysis to identify key active components.

### 4.6. Molecular Docking

According to the methods described in the literature [52,53], key active components and core targets identified from network pharmacology and PPI analysis were subjected to molecular docking. Small-molecule ligands (active components) were obtained from PubChem (https://pubchem.ncbi.nlm.nih.gov/, available at 10 April 2025), and minimized using ChemBio3D v23.1.1, converted to PDB format. Protein structures were retrieved from PDB (https://www1.rcsb.org/, available at 10 April 2025), processed using PyMOL v3.1.6, and docked with AutoDock Vina v1.2.5 to obtain the optimal binding conformations. The docking results were visualized using Discovery Studios 2019.

### 4.7. In Vitro Lipid-Lowering Activity Evaluation of BHEE

#### 4.7.1. Cell Culture

HepG2 cells (purchased from Procell Life Science & Technology Co., Ltd., Wuhan, China, Cat. No. CL-0103. Passage number: 8) were cultured in HepG2-specific medium (Procell Life Science & Technology Co., Ltd., Wuhan, China, Cat. No. CM-0103) containing 10% FBS and 1% antibiotics (penicillin/streptomycin) at 37 °C, 5% CO_2_. The medium was replaced every 24 h, and cells were passaged when they reached 80% confluence.

#### 4.7.2. CCK-8 Assay for Cell Viability

HepG2 cells were seeded in 96-well plates and incubated overnight. After discarding the medium, different concentrations of BHEE (25, 50, 100, 200, 400, 800, 1000 mg·mL^−1^) were added, and cells were incubated for 24 h. Cell viability was measured using CCK-8 (Elabscience Biotechnology Co., Ltd., Wuhan, China, Cat. No. E-CK-A362) reagent at 450 nm. The formula for calculating cell viability is provided.

#### 4.7.3. High-Fat Cell Model Construction and Treatment

FFA-induced HepG2 cell models were established by treating cells with a mixture of oleic acid and palmitic acid (0.25 mM and 0.5 mM, respectively) (Xi’an Kunchuang Technology Development Co., Ltd., Xi’an, China, Cat. No. KC006) for 24 h. Cells were then divided into control, model, low-dose (BHEE-L, 25 mg·mL^−1^), medium-dose (BHEE-M, 50 mg·mL^−1^), and high-dose (BHEE-H, 100 mg·mL^−1^) groups.

#### 4.7.4. Lipid Accumulation, Oxidative Stress, and Inflammation Detection

Oil Red O staining was used to observe lipid droplets. Image analysis was performed using Fiji v2.14.0 software, and the relative area of lipid droplets was calculated as the ratio of the “total lipid droplet area to total cell area” within the field of view. The final data were derived from three independent experiments. TG (Cat. No. A110-1-1, intra-assay CV ≤ 5%, inter-assay CV ≤ 8%), TC (Cat. No. A111-1-1, intra-assay CV ≤ 3%, inter-assay CV ≤ 5%), LDL-C (Cat. No. A113-1-1, intra-assay CV ≤ 8%, inter-assay CV ≤ 10%), HDL-C (Cat. No. A112-1-1, intra-assay CV ≤ 3%, inter-assay CV ≤ 5%), CAT (Cat. No. A007-1-1), GSH (Cat. No. A006-2-1, intra-assay CV: 1.2%, inter-assay CV: 3.86%), SOD (Cat. No. A001-3, intra-assay CV: 5.05%, inter-assay CV: 3.32%), and MDA (Cat. No. A003-4-1) levels were measured using the corresponding kits from Nanjing Jiancheng Bioengineering Institute (Nanjing, China). TNF-α (Cat. No. E-EL-H0109, intra- and inter-assay CV < 10%), IL-6 (Cat. No. E-EL-H6156, intra- and inter-assay CV < 10%), and MMP9 (Cat. No. KE00456, intra- and inter-assay CV < 10%) levels were determined using ELISA kits from Elabscience Biotechnology Co., Ltd. (Wuhan, China), and Wuhan Sanying Biotechnology Co., Ltd. (Wuhan, China), respectively.

### 4.8. Statistical Analysis

Data were analyzed using GraphPad Prism 10.4.1 software. Results are expressed as mean ± standard deviation (SD). One-way ANOVA followed by Tukey’s post hoc test was used for multiple comparisons, and *p* < 0.05 was considered statistically significant.

## 5. Conclusions

The findings of this study suggest that *B. heterostemon* may exert effects on multiple targets, likely through a multi-component mechanism. However, further experimental validation is necessary to determine whether these effects are additive, synergistic, or driven by a single, key compound. While our network pharmacology analysis supports the hypothesis of a multi-target action, the precise mechanisms underlying BHEE’s effects remain to be confirmed in future studies. In future research, we plan to evaluate the in vivo efficacy of BHEE using animal models, such as high-fat-diet-induced hyperlipidemic mice, and to validate the regulation of key targets and pathways through techniques like Western blot and qPCR. These studies will help elucidate the molecular mechanisms of BHEE, providing a stronger foundation for its development as a natural lipid-lowering agent.

## Figures and Tables

**Table 1:** Active components of BHEE and their absorption parameters.

No	Name	GI Absorption	Number of Drug-Likeness Criteria Met	No	Name	GI Absorption	Number of Drug-Likeness Criteria Met
M1	protopine	High	5	M26	medioresil	High	5
M2	luteolin	High	3	M27	2-phenylacetamide	High	5
M3	6-hydroxyluteolin	High	5	M28	hydroxysanguinarine	High	5
M4	6-hydroxycoumarin	High	5	M29	ferulic acid	High	3
M5	malic acid	High	3	M30	tectorigenin	High	3
M6	eupatorin	High	3	M31	succinic acid	High	4
M7	irigenin	High	5	M32	iristectorigenin B	High	5
M8	iristectorigenin A	High	5	M33	traumatic acid	High	3
M9	skimmin	High	5	M34	undecanedioic acid	High	5
M10	isololiolide	High	4	M35	(S)-abscisic acid	High	5
M11	skullcapflavone II	High	4	M36	epipodophyllotoxin	High	5
M12	umbelliferone	High	5	M37	sebacic acid	High	5
M13	Viscidulin I	High	3	M38	2-hydroxy-3-methylpentanoic acid	High	5
M14	5,6,7,4′-tetrahydroxy-8-methoxyisoflavone	High	5	M39	granilin	High	5
M15	2,5-dihydroxy-1-methoxy-anthraquinone	High	5	M40	scopoletin	High	3
M16	ganhuangenin	High	5	M41	n-trans-feruloylmethoxytyramine	High	5
M17	flazin	High	5	M42	5-hydroxy-3,6,7,4′-tetramethoxyflavone	High	3
M18	4-formyl indole	High	5	M43	3-acetoxy-5,7-dihydroxyflavanone	High	5
M19	cirsimaritin	High	3	M44	3-methylherbacetin	High	5
M20	cis-p-coumaric acid	High	5	M45	3-hydroxypalmitic acid	High	5
M21	dihydroactinidiolide	High	3	M46	6-methyl-7-(3-oxobutyl)bicyclo [4.1.0]heptan-3-one	High	5
M22	alismoxide	High	3	M47	(2R)-2-butoxybutanedioic acid	High	3
M23	azelaic acid	High	4	M48	dehydrovomifoliol	High	4
M24	6-methoxytricin	High	5	M49	vulgarin	High	4
M25	casticin	High	5	M50	cadinanetriol	High	5

**Table 2:** Key Targets and Their Topological Parameters.

Target	Degree	Betweenness	Closeness
TNF	103	2158.37	0.77
IL6	103	1919.52	0.77
AKT1	94	1619.33	0.74
PPARG	81	1448.58	0.69
EGFR	67	726.301	0.65
NFκB1	67	346.51	0.64
ESR1	66	506.02	0.64
CTNNB1	62	562.28	0.63
HSP90AA1	60	445.15	0.63
MMP9	58	207.21	0.62

**Table 3:** Topological parameters and classifications of key active components.

Key Active Components	Degree	Betweenness	Closeness	Classification
6-methoxytricin	27	261.34	0.41	Flavonoids
eupatorin	26	220.18	0.40	Flavonoids
ganhuangenin	26	277.52	0.41	Flavonoids
flazin	26	619.32	0.41	Alkaloids
vulgarin	26	565.62	0.41	Terpenes
luteolin	25	216.27	0.41	Flavonoids
6-hydroxyluteolin	25	218.48	0.41	Flavonoids
skullcapflavone II	25	342.86	0.40	Flavonoids
cirsimaritin	25	251.58	0.40	Flavonoids
casticin	25	215.21	0.41	Flavonoids

## Data Availability

The datasets analyzed as part of this study are obtainable in the TCM Integrated Database (TCMID, https://www.bidd.group/TCMID/, available at 15 March 2025), PubChem (https://pubchem.ncbi.nlm.nih.gov/, available at 20 March 2025), Swiss ADME (http://www.swissadme.ch/, available at 25 March 2025), SwissTargetPrediction (http://swisstargetprediction.ch/, available at 27 March 2025), GeneCards (https://www.genecards.org/, available at 2 April 2025), OMIM (https://omim.org/, available at 2 April 2025), DrugBank (https://go.drugbank.com/, available at 2 April 2025), Uniport (https://www.uniprot.org/, available at 2 April 2025), online platform Bioinformatics (http://www.bioinformatics.com.cn/, available at 2 April 2025), STRING database (https://cn.string-db.org/, available at 4 April 2025), DAVID (https://david.ncifcrf.gov/, available at 5 April 2025), Protein Data Bank (PDB, https://www1.rcsb.org/, available at 10 April 2025).

## Supplementary Materials

The following supporting information can be downloaded at: https://www.mdpi.com/article/10.3390/plants14223535/s1, Table S1: Qualitative analysis of compounds in the ethyl acetate extract of *Biebersteinia heterostemon*; Table S2: Active ingredient screening; Table S3: Target prediction of active ingredients in BHEE; Table S4: Targets of hyperlipidemia were predicted using the GeneCards Database, Online Mendelian Inheritance in Man Database (OMIM) and DrugBank Database; Table S5: Common targets between the potential targets of active ingredients from BHEE and the pathology-related targets of hyperlipidemia; Table S6: Attribute values of the common target PPI network; Table S7: GO functional annotation of common targets; Table S8: KEGG enrichment analysis of common targets; Table S9: “Drug-Effective Component-Intersecting Target-Pathway” interaction network; Figure S1: Total ion chromatogram (TIC) of ethyl acetate extract fraction from *Biebersteinia heterostemon* via UHPLC-Q-Exactive-Orbitrap-MS/MS; Figure S2: Intersection target protein–protein interaction (PPI) Network. Figure S3: Molecular docking analysis of key active components and core targets of BHEE in improving hyperlipidemia.

## Abbreviations

The following abbreviations are used in this manuscript:

BP	Biological Process
CC	Cellular Component
FFA	Free Fatty Acid
FBS	Fetal Bovine Serum
GO	Gene Ontology
KEGG	Kyoto Encyclopedia of Genes and Genomes
MF	Molecular Function
PPI	Protein–Protein Interaction
HLP	Hyperlipidemia
BHEE	Ethyl Acetate Extract of *B. heterostemon*
CCK-8	Cell Counting Kit-8
