# Supplementary material for: Unraveling the Mechanisms of Biebersteinia heterostemon in Improving Hyperlipidemia: A Network Pharmacology, Molecular Docking, and In Vitro Validation in HepG2 Cells
Source: Plants (Basel). 2025 Nov 19;14(22):3535. doi: 10.3390/plants14223535 (PMC12656387; doi:10.3390/plants14223535)
Supplement: Supplementary file 1 [file plants-14-03535-s001.zip › Figures S1-S3.pdf]

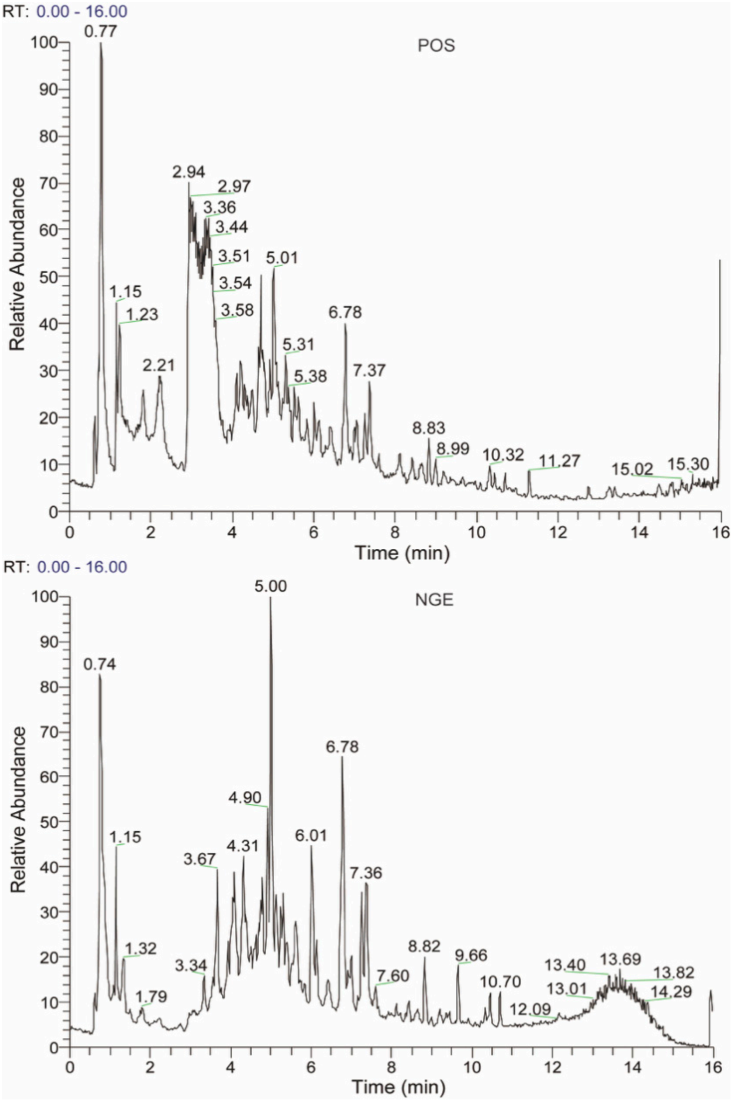

Figure S1. Total ion chromatogram (TIC) of ethyl acetate extract fraction from *Biebersteinia heterostemon* via UHPLC-Q-Exactive-Orbitrap-MS/MS. (A) TIC in positive ion mode. (B) TIC in negative ion mode.

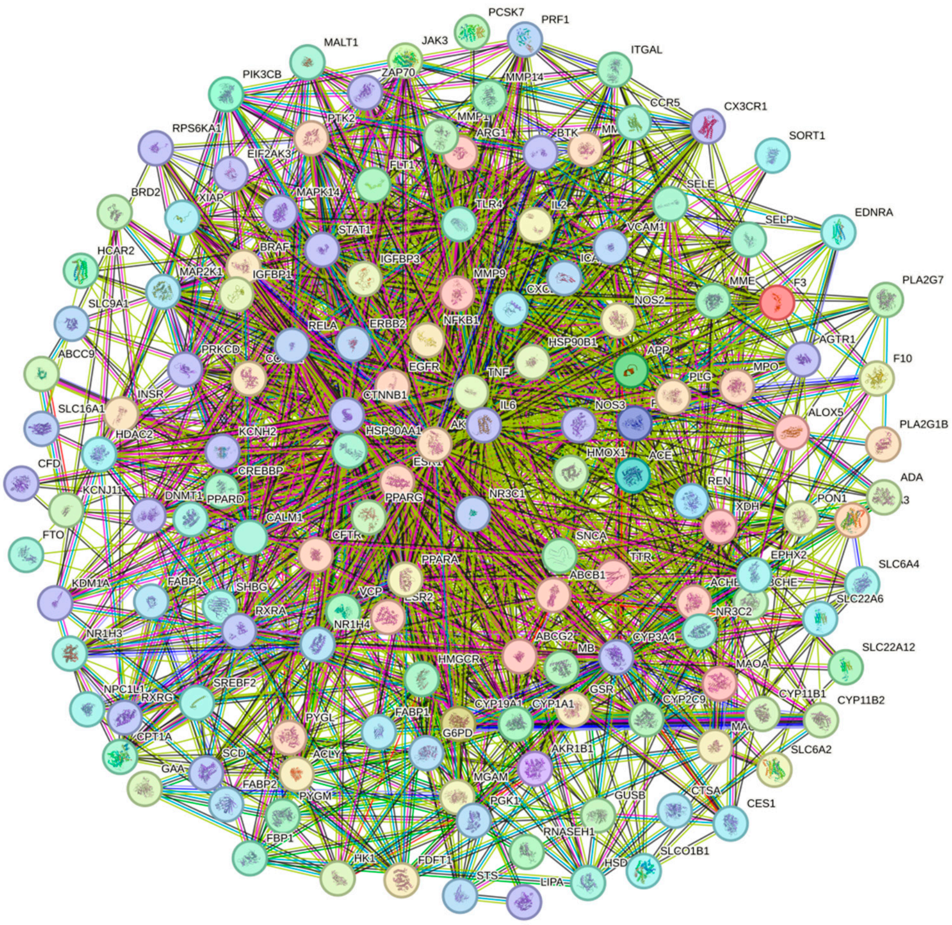

Figure S2. Intersection target protein-protein interaction (PPI) Network.

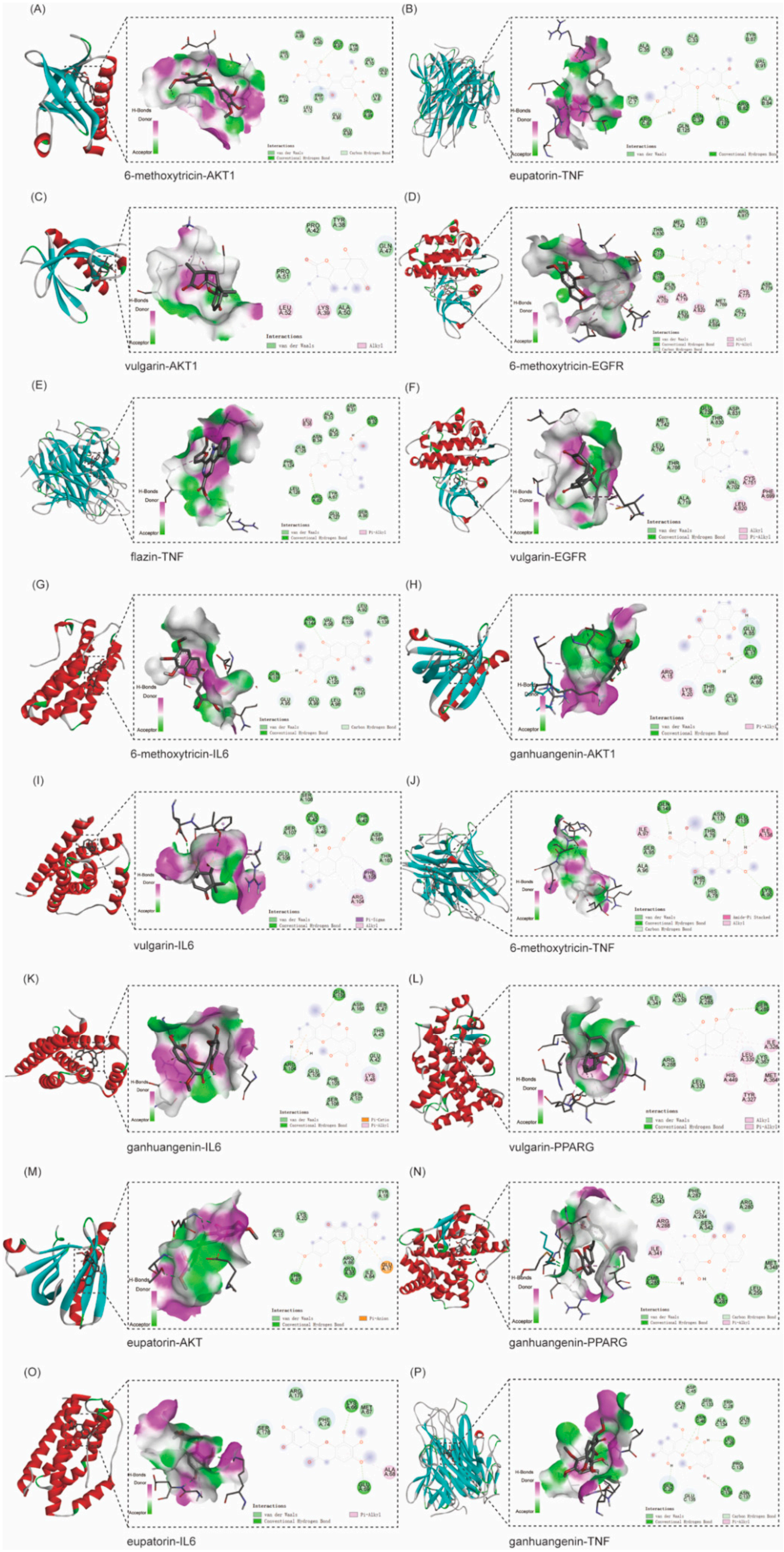

Figure S3. Molecular docking analysis of key active components and core targets of BHEE in improving hyperlipidemia. (A) Docking of 6-methoxytricin with AKT1. (B) Docking of eupatorin with TNF. (C) Docking of vulgarin with AKT1. (D) Docking of 6-methoxytricin with EGFR. (E) Docking

of flazin with TNF. (F) Docking of vulgarin with EGFR. (G) Docking of 6-methoxytricin with IL6. (H) Docking of ganhuangenin with AKT1. (I) Docking of vulgarin with IL6. (J) Docking of 6-methoxytricin with TNF. (K) Docking of ganhuangenin with IL6. (L) Docking of vulgarin with PPARG. (M) Docking of eupatorin with AKT1. (N) Docking of ganhuangenin with PPARG. (O) Docking of eupatorin with IL6. (P) Docking of ganhuangenin with TNF.
